# Supplementary material for: Causal Relationship between Aging and Anorexia Nervosa: A White-Matter-Microstructure-Mediated Mendelian Randomization Analysis
Source: Biomedicines. 2024 Aug 16;12(8):1874. doi: 10.3390/biomedicines12081874 (PMC11351342; doi:10.3390/biomedicines12081874)
Supplement: Supplementary file 1 [file biomedicines-12-01874-s001.zip › Supplementary Figure/Figure S7/Single SNP plot of MR analysis for ubm-b-1950 to AN.pdf]

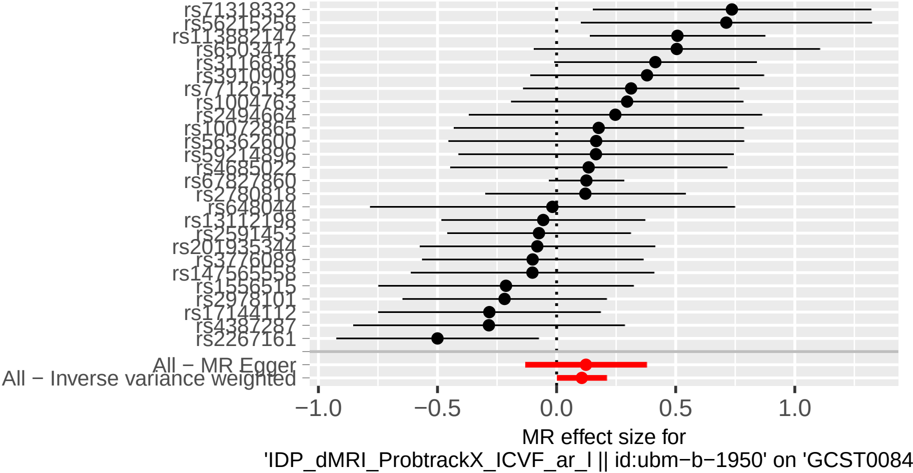
rs71318332
rs56215258
rs113882147
rs6503412
rs3116836
rs3910909
rs77126132
rs1004763
rs2494664
rs10072865
rs56362600
rs59214896
rs4685022
rs67827860
rs2780818
rs648044
rs13112198
rs2591453
rs201935344
rs3776089
rs147565558
rs1556515
rs2978101
rs17144112
rs4387287
rs2267161
All − MR Egger
All − Inverse variance weighted
−1.0
−0.5
0.0
0.5
1.0
MR effect size for
'IDP_dMRI_ProbtrackX_ICVF_ar_l || id:ubm−b−1950' on 'GCST0084
